# Perception of Japanese singleton and geminate contrasts: A case of Chinese learners with different dialectal backgrounds

**DOI:** 10.3389/fpsyg.2022.1070107

**Published:** 2022-12-07

**Authors:** Honghao Ren

**Affiliations:** School of Foreign Studies, Xi’an Jiaotong University, Xi’an, China

**Keywords:** cross-linguistic speech perception, Japanese singleton and geminate contrasts, dialectal background, Chinese learners, positive transfer

## Abstract

It is widely accepted that the Japanese language is mora-timed, and the geminate obstruent, one of the three special morae in the Japanese language, forms one independent mora. Many studies have shown that perceiving Japanese geminates accurately is especially problematic for learners of Japanese. This study examines the perception of Japanese singleton and geminate contrasts by Chinese learners of Japanese (CLJ) from different dialectal backgrounds and contrasts their perception with Japanese native speakers (JNS). This study conducted two experiments. First, non-synthesized stimuli were used to test each group of participants’ perceptual sensitivity of Japanese singletons and geminates. Second, the categorical perception was accessed by adopting stimuli in which the ratio of constriction duration to entire word duration (CD/EWD) was synthesized from 15 to 60%. Results show that, although learners of Japanese had significantly lower perception levels compared to JNS regardless of their group differences (e.g., L1 background, Japanese language proficiency, etc.), they still experienced some positive transfers of L1 to achieve better perception performance. The results also suggest that CD/EWD can be considered a reliable local cue for both JNS and CLJ in categorizing Japanese singletons and geminates. In addition, the results demonstrate that variables such as medial consonant type and the learner’s Japanese proficiency affect the perception of Japanese singleton and geminate contrasts.

## Introduction

Second language acquisition (SLA), especially second language (L2) speech acquisition, has been widely discussed for decades. During this process, several learning models efficiently predicted and explained learners’ biases and tendencies in L2 speech acquisition, such as perceptual assimilation model (PAM) (Best, 1995; Best et al., 2001) and speech learning model (SLM) (Flege, 1995). Producing and perceiving L2 speech at a native-like level is considered an ongoing struggle for learners who are experiencing difficulties in L2 acquisition. However, naïve learners also show different difficulty levels for non-native contrasts due to factors such as phonetic distance between L1 and L2 segments (e.g., Best, 1995; Flege, 1995), degrees of experience with the L2 (e.g., MacKay et al., 2001; Flege and MacKay, 2004), etc.

Another perspective is how language transfer could account for mapping the characteristics of L2 speech acquisition. It is widely agreed that L2 acquisition could be strongly influenced by the learner’s native language (L1) or dialect. It has also been stated that aspects of phonology show significant evidence of language transfer (Norris and Ortega, 2000; Toda, 2001; De Angelis, 2007). Therefore, making use of positive L1 transfers and avoiding negative ones may improve the acquisition of the target language. Previous studies have also found complex interactions between learners’ linguistic experience as well as the phonological system of their L1 and the identification of L2 phonological categories (Cutler, 2001; Strange, 2011; Chang, 2018). Numerous studies have confirmed that contrasts that are absent in learners’ L1 may present challenges for L2 acquisition. For example, McAllister et al. (2002) compared the acquisition of Swedish quantities by native speakers of American English, Latin American Spanish, and Estonian. Results showed that native Estonian speakers much closely matched native Swedish speakers. This could be considered as evidence that the presence of quantity distinctions based on the duration feature in Estonian appears to have a positive transfer in native Estonian speakers’ learning of Swedish quantity distinctions. It also showed that the native English subjects were more successful in learning Swedish quantity contrasts than the native Spanish subjects. The result would also reflect the prominence of the positive transfer of the duration feature in the L1 due to the fact that the duration feature plays a more distinct role in English than in Spanish. Tsukada et al. (2018) demonstrated that native Japanese and Italian speakers showed better perception on the processing of consonant length in an unknown language over Australian and Korean learners of Japanese whose L1 lacks long and short consonant contrast. This may suggest that previous experience with consonant length in native Japanese and Italian speakers’ L1 facilitated their perception of singletons and geminates. Although there are several studies investigating the effects of L1 phonology and phonetics on L2 phonemic contrasts, the experimental evidence on Chinese, especially non-Mandarin as a first language, versus Japanese as a target language is sporadic. Therefore, it is important to examine the effects of L1 phonology and phonetics on L2 phonemic contrasts in this study.

Japanese is known as a typical mora-timed language, while Chinese is a syllable-timed one. A mora is a phonologic unit that determines timing in some languages, including Japanese, and it is used as the basis of the sound system. There are three special morae in the Japanese language (i.e., obstruent geminate, moraic nasal, long vowel) and they reveal the characteristics of Japanese phonology. As Chinese is not a mora-timed language, it can be difficult for learners of Japanese to understand this concept. Producing and perceiving Japanese special morae accurately is a common problem among learners of Japanese. Moraic nasals and long vowels in Japanese language usually do not present as much of a challenge, as learners of Japanese can perceive and produce them with the assistance of similar sounds in their L1. However, the short-long consonant contrast only appears in some Chinese southern dialects, such as Cantonese, Fukienese, and Hakka, which is a homorganic consonant sequence that can occur at a word boundary rather than occur as contrastive geminates. Thus, speech acquisition of Japanese geminate consonants (*Sokuon* in Japanese) is likely to be particularly problematic for learners of Japanese, such as for Mandarin-speaking learners.

When learning L2 speech, perceiving and producing sound contrasts are two necessary processes that L2 learners have to face. The relationship between speech perception and production has been a controversial topic under ongoing debate. Although it has not reached an agreement on how the interaction between perception and production occurs, most researchers have claimed a close connection between these two modalities (e.g., Liberman et al., 1967; Liberman and Mattingly, 1985; Fowler, 1986). Within these studies, it is widely acknowledged that speech perception takes precedence over speech production and affects the performance of speech production to a certain extent. For example, Barry (1989) suggested that L2 learners who are able to detect sound categories better also have less difficulty in L2 speech production. Flege (1995) claimed that non-native speech categories are firstly carried out in speech perception, and learners can adopt those same categories when producing L2 speech. Speech perception involves different areas of the brain compared with those used in production (Obleser and Eisner, 2009). This means that speech perception has unique characteristics and complexity, which differs from speech production. Therefore, it is important to gain a better understanding of the way learners of Japanese perceive Japanese singleton-geminate contrasts.

There has been some research on the perception of Japanese geminate consonants, taking both JNS and learners of Japanese as subjects. Previous studies on the perception of Japanese geminate consonants put more focus on the perceptual cues for singleton-geminate identification. Research generally agrees that the constriction duration difference between singleton and geminate is the primary acoustic cue (Fujisaki et al., 1975; Fujisaki and Sugito, 1977; Otsubo, 1981; Lahiri and Jorge, 1988; Hankamer et al., 1989; Abramson, 1991; Idemaru and Guion, 2008; Ridouane, 2010). Fujisaki and Sugito (1977) examined the wide-band spectrogram of minimal pairs with different meanings, such as [*ise*] “Ise (place name)” versus [*is:e*] “one ridge.” They found that the friction duration (FD) in fricatives /s/ versus /ss/ and the closure duration (CD) in plosive /t/ versus /tt/ was a definite acoustic correlate when distinguishing words with singleton-geminate consonants. They also concluded that the characteristics of consonant duration in Japanese words play an important role in the transmission of linguistic information. Furthermore, Otsubo (1981) adopted nonsense words in CV(C)CV (/bapa/ vs. /bappa/) as stimuli to conduct an identification task. This study concluded that despite the proximate sound environment showing some influence on the perception of singleton-geminate words, JNS still takes closure duration of plosive /p/ as the most reliable cue in categorizing singleton and geminate stops.

For the perception of singleton and geminate contrasts, several other durational correlates have also been proposed as secondary cues. Watanabe and Hirato (1985) analyzed how native speakers of the Tokyo dialect perceive /appa/, /atta/, /akka/ contrasts with various speech rates and synthesized sounds with the closure duration shortened or lengthened. This study reported that the length of vowels neighboring geminate consonants affects the perceptual boundary point in the categorical perception of Japanese singleton and geminate contrasts. It is therefore clear that in a CV_1_(C)V_2_ sequence, there is a correlation between V_1_ duration and the perceptual boundary point of singleton-geminate contrasts, although researchers still could not reach an agreement on whether this correlation is positive or negative. For example, Watanabe and Hirato (1985) and Hirata (1990) suggested that a longer extended V_1_ leads to a greater value of perceptual boundary point, while Ofuka et al. (2005) found that contrasts with longer V_1_ were easier to be perceived as geminates. These results confirm the preceding vowel length as a secondary correlate.

Of course, a singleton-geminate contrast is perceived not only in raw durations but also in terms of relational timing cues, which are considered more stable. Fujisaki and Sugito (1977) explored the perception of 2- versus 3-mora contrasts (/ise/ vs. /isse/, /ita/ vs. /itta/) employing synthesized stimuli. They found that once the ratio of friction duration to the preceding vowel duration is 1.66 or more, or the ratio of closure duration to the preceding vowel duration is above 1.69, one stimulus is more likely to be perceived as a geminate word. This study also revealed that the perception of a geminate consonant correlates with the duration of its preceding mora. Idemaru and Guion (2010) successfully used synthesized sounds based on /seta/ to conduct a perception task and found that both the ratio of closure duration to preceding mora duration (C/M_1_) and the ratio of closure duration to the following vowel duration (C/V_2_) are stable cues in perceiving singleton and geminate contrasts. Additionally, C/M_1_ is reported to have a more significant discrimination effect than C/V_2_.

Prior studies on the perception of Japanese gemination have also explored the non-durational cues that affect how well speech is perceived (Hirata, 1990; Ofuka, 2003; Kubozono et al., 2011; Yanagisawa and Arai, 2015; Zhang, 2019, 2020). Ofuka (2003) tested whether accent patterns affect the perception of singleton-geminate contrasts using synthesized stimuli of the words[*kata*] “shoulder” versus [*kat:a*] “won,” which have a high(-low)-low < H(L)L > accent pattern, and [*kata*] “type” versus [*kat:a*] “bought,” which have a low(-high)-high < L(H)H > pattern. This study found that the value of perceptual boundary point of the stimuli with L(H)H pattern is significantly higher than those with H(L)L. Hirata (1990) produced the same results, suggesting that the accent pattern can be considered a secondary cue of perception of Japanese singleton and geminate pairs. Furthermore, Yanagisawa and Arai (2015) presented JNS with synthesized stimuli with the preceding vowel ranked along a 10-step intensity scale, two steps (with formant transitions and without transitions) of F1 and F2, as well as a 15-step closure duration continuum ranging from 100 to 380 ms. Among the stimulus groups with formant transitions, F1 was varied from 750 to 500 Hz and F2 from 1,250 to 1,400 Hz. The results showed that formant transitions strongly influence the perception of Japanese geminate consonants, while the damping of intensity did not significantly affect perception.

### Study aims

The above studies on Japanese singleton-geminate contrasts indicate that the perception of Japanese singleton and geminate consonants may be affected by a number of factors, such as constriction duration, neighboring vowel types, and acoustic transitions. However, the majority of this research does not focus on the perception of learners of Japanese, nor is it clear to what extent L1 transfer occurs with respect to the perception of Japanese singleton and geminate contrasts. Specifically, it has been attested that some Chinese dialects’ phonological features positively influence the production of Japanese singleton and geminate contrasts (Wang, 1999; Zhang et al., 2015; Ren and Kondo, 2020). Checked tone, commonly known by the Chinese calque entering tone, is a syllable that ends in a stop consonant (e.g., [t], [d], [k], [g], [ʔ]). Tones are always related to pitch or pitch patterns such as high rising, low falling, etc. Readers may wonder why we mention syllable finals here when discussing checked tones. Indeed, it is called a “tone,” but the main characteristic of its tone value is the *shortness* in length, which is in contrast to that of lax tone. According to Matthews and Yip (2011, p. 29), /sit/ “lose” and /si/ “matter” are phonetically the same in tone level, differing only in length. In other words, it is a chroneme due to the long/short contrasts of sound rather than a toneme formed by different pitch changes (Yang, 1997). Checked tone is only present in some Chinese southern dialects, and this syllable structure does not appear in the phonological system of Mandarin. As one of the Chinese southern dialects, Cantonese is a more complex tonal language than Mandarin. Three stops (i.e., /p/, /t/, /k/) could appear in Cantonese checked tones, meaning only those unreleased consonants can be used as syllable finals as a segmental characteristic. These ‘cat tail’ type consonants are not underlying geminates but can somehow derive long consonant sequences in Cantonese (Lee and Mok, 2018). For example, if the final consonant of a syllable is the same as the initial one of the following syllable (e.g., [*zok ke*] “be a guest”), a long consonant structure that is similar to *Sokuon* in Japanese is formed.

Therefore, three research questions were posed in the current study as follows.

(1) Do the use of long and short consonants in learners’ L1 help their perception of Japanese singleton/geminate consonants?

In Ren and Kondo (2018), a priori study, we recruited 41 Cantonese-speaking learners of Japanese to analyze the perception of Japanese singleton/geminate contrasts by Cantonese-speaking learners of Japanese. The study concluded that the use of long and short consonants in learners’ L1 was positively transferred to their perception of Japanese gemination by comparing the perceptual identification accuracy and categorical perception boundary points in different consonant conditions. More specifically, interesting possibility can be raised that Cantonese-speaking learners of Japanese may have an advantage over Mandarin-speaking learners and Japanese singleton/geminate contrasts may be perceived with different difficulty levels. For example, differing from plosives /p/, /t/, and /k/, fricative /s/ can never be used as syllable-final, which means that [s:] does not occur in Cantonese neither phonetically nor phonologically. This makes it predictable that Cantonese-speaking learners may therefore perform less native-like of Japanese VsV-VssV distinction than other V(C)CV sequences with plosives (e.g., VpV-VppV, VkV-VkkV, etc.). To better prove this hypothesis, we recruited an additional 43 learners of Japanese from two different L1 backgrounds (see Table 1) to conduct this study.

**TABLE 1:** Overall characteristics of participants in the perception experiment.

Group		Gender	Age	Holders of JLPT	Learning period (Months)
JNS		M: 5; F: 5	18–22 (Avg. = 20.70, *SD* = 1.34)	–	–
MSL	Beginner	M: 4; F: 8	18–21 (Avg. = 19.83, *SD* = 0.94)	N3: 1 N/A: 11	12–38 (Avg. = 19.83, *SD* = 6.78)
	Advanced	M: 4; F: 6	25–27 (Avg. = 25.60, *SD* = 0.70)	N1: 10	41–96 (Avg. = 77.40, *SD* = 15.83)
CSL	Beginner	M: 1; F: 10	18–20 (Avg. = 19.36, *SD* = 0.67)	N3: 1 N/A: 10	11–15 (Avg. = 12.55, *SD* = 1.04)
	Advanced	M: 5; F: 5	21–22 (Avg. = 21.40, *SD* = 0.52)	N1: 10	36–42 (Avg. = 36.70, *SD* = 1.89)

In defining learners’ Japanese proficiency, we are accustomed to relying on the results of the Japanese-language proficiency test (JLPT). Although this is sometimes effective, the JLPT is more inclined to judge a learner’s vocabulary, grammar, and reading ability, making it challenging to comprehensively rate learners’ speech acquisition level by this test alone. Therefore, learner-oriented multiple measurements were used in this study to differentiate learners’ overall Japanese language ability, as mentioned above. Thus, the number of “N/A” under the column “Holders of JLPT” represents the number of beginners who are not JLPT certified.

(2) Are there additional acoustic cues other than those mentioned above that impact the perception of Japanese geminate consonants?

Besides vocalic parameters, there is a reason to believe that perception would correspondingly differ with the change of medial consonant manner of articulation itself. When selecting medial consonants, we considered which singleton and geminate contrasts are more difficult for learners of Japanese to perceive. Kawahara (2015) showed the results of duration measurements of singleton and geminate consonants based on data of three JNS. The results indicate that singleton and geminate duration ratios of /p/ and /k/ are the lowest for plosives. The duration ratio for fricative /s/ is similarly low, but the perception of Japanese gemination with /s/ as medial consonant is rarely discussed. This indicates that singleton and geminate contrasts with these medial consonants may be less perceptible for learners of Japanese and need further analysis. Thus, several relational correlates have been proposed in distinguishing Japanese singleton and geminate contrasts, such as C/V_1_ ratio (Hirata and Whiton, 2005; Idemaru and Guion, 2010), V-to-V interval (Hirata and Forbes, 2007), C/Mora ratio (Hirata and Forbes, 2007; Idemaru and Guion, 2010), and C/W ratio (Hirata and Whiton, 2005; Idemaru and Guion, 2010; Ren and Kondo, 2020). If relational timing consistently categorizes the perception of Japanese singleton and geminate contrasts, it would provide strong support for its likelihood as a primary perceptual cue among learners of Japanese. Therefore, ratio of constriction duration to entire word duration (CD/EWD) is used as a relational measure that focuses on the primary acoustic cue (constriction duration) as well as taking its relative proportion into account.

(3) Does the Japanese proficiency of learners of Japanese affect their perception of singleton and geminate contrasts?

It is generally believed that the level of L2 acquisition is associated with L2 experience and increases with the progress of learning. Previous research suggest that increased L2 experience can lead to an improvement in L2 perception (Tees and Werker, 1984; Flege et al., 1997; Levy, 2009). However, when L1 background and L2 experience come to individuals simultaneously, more experienced L2 learners do not always have better L2 perception; in fact, L2 experience may even hinder performance in perceptual tasks (Flege et al., 2006; Aoyama et al., 2008; Holliday, 2015, 2016).

We designed two mutually independent perceptual experiments to address the above research questions. The goal of experiment 1 was to explore the way learners perceive non-synthesized singleton and geminate contrasts. Specifically, this experiment analyzed the perceptual differences between learners of Japanese from two dialectal backgrounds and compared them to that of a JNS control group. Perception experiment 2 adopted synthesized sounds with the ratio of constriction duration to entire word duration (CD/EWD) expanded or contracted. Identification tasks via two-alternative forced-choice (2AFC) were conducted to assess the categorical perception of Japanese singleton and geminate contrasts and the difference between groups.

## Perception experiment 1

### Methods

#### Participants

The participants comprised 53 adult perceivers, while two JNS (one male and one female) served as speakers whose utterances were used to create speech stimuli used in this perception experiment. These two voice providers were Tokyo dialect speakers from the Kanto area. They both work in the field of Japanese language education and are knowledgeable in phonetics and phonology. The perceivers were divided into three groups based on their linguistic background: the Japanese native speaker group (JNS), the Mandarin-speaking learner group (MSL), and the Cantonese-speaking learner group (CSL).

Ten native speakers of the Tokyo Japanese dialect (five males and five females) were recruited from among undergraduate students at Waseda University in Tokyo, Japan. A total of 22 MSLs and 21 CSLs participated. The MSLs and CSLs were further subdivided into two groups–beginner and advanced–based on their Japanese proficiencies. All MSLs and CSLs were regular undergraduate or graduate students at universities in China at the time of testing. As a speech perception study, it is challenging for the current study to define a learner’s Japanese proficiency based on tests such as the JLPT alone, especially for research concerning learners’ speech acquisition. Therefore, we adopted multiple measurements, including the JLPT score, learning period, and supervisor appraisal, to evaluate learners’ Japanese proficiency as comprehensively and objectively as possible. We first classified learners into beginners and advanced according to the JLPT results and the Japanese language learner period. Then we asked the teachers who were in charge of their comprehensive Japanese course (most familiar with students’ learning situation of the Japanese language) to carry out a four-point-scale appraisal (i.e., beginner, intermediate, pre-advanced, advanced) on the participants’ Japanese proficiency referring to the participants’ Japanese-related academic performance (e.g., periodic test scores). Only learners whose two-stage assessments were aligned as a beginner or advanced, respectively, were employed as participants. In addition, MSLs come from universities in Dalian and Beijing, and CSLs are from a university in Guangzhou, China. All groups of perceivers were compensated for their participation and all participants were asked to confirm that they had no hearing disorders. None of the perceivers reported having experience studying or residing abroad for over half a year.

A reading aloud test (RAT) was done using Chinese dialect versions of phonetically balanced material: *The north wind and the sun*. The entire test process was recorded, and 4 (two for each dialect) proficient dialect speakers with more than 20 years of dialect experience were invited to evaluate the reading aloud materials. This is to confirm whether MSLs and CSLs have the ability to read aloud fluently and use their dialects freely. Only those who are with this ability were selected as subjects for this study. Table 1 shows the detailed information on the participants. Thus, we got the appropriate ethical approval for this study, and all participants gave their informed consent for this study.

#### Stimuli

As mentioned above, there is evidence showing that speech perception can be influenced by the neighboring acoustic environment. For this reason, the preceding and following vowels of test words were limited to three types: low /a/, mid /e/, and high /i/. The medial consonants selected were plosive /k/ and /p/ and fricative /s/. In total, nine minimal pairs with two syllables, in which one contains a geminate and another a singleton, were employed as test words (e.g., /aka/ vs. /akka/, /ipi/ vs. /ippi/, etc.). All of the test words were initial-accented (HL or HLL) words^1^.

The 18 test words (see Supplementary Appendix 1) were produced by two Japanese native speakers, six times at a natural tempo and digitally recorded. Stimuli were embedded in a carrier sentence, “*Sore wa (test word) desu.*,” meaning “*That is (test word).*” As mentioned above, test words used in this study are all in V(C)CV sequence. Due to this, the two voice providers were asked to slightly make a pause between the “*wa*” and the test word. This can make the initial of all test words clear and distinguished from the carrier phrase, especially for those in the “*a(C)Ca*” sequence. Recordings were done in a soundproof studio at a university in Tokyo with a SONY F-780 microphone connected to a digital sound recorder (MarantzPMD 561), with 16-bit quantization at 44.1 kHz sampling frequency. The recording pace was controlled by the researcher. A total of 216 tokens (18 test words × 2 speakers × 6 repetitions) were recorded. Thus, this study invoked the segmentation criteria used in Fischer-Jørgensen and Hutters (1981). For each speaker, three utterances without any editing were selected for each test word and only the test word sections in these utterances (totally 108: 18 test words × 3 selected utterances × 2 speakers) were segmented from carrier sentences as stimuli^2^. Then, stimuli used in perception experiment 1 (see details in Supplementary Appendix 2) were created by adding 500-ms white noises before and after each test word with the amplitude normalized to 65 dB. This is to make the presentation of the stimuli less abrupt for subjects and to ensure the naturalness of each stimulus.

#### Procedures

Perception experiment 1 was conducted using the ExperimentMFC function of Praat (version 6.0.34). The experiment was conducted on different days depending on the group of subjects. All experiments were conducted one-on-one, with the participants tested individually in a quiet multimedia room at universities in Japan and China. Each participant was asked to sit in front of a monitor, and the stimuli were presented to them over wireless circumaural headphones (SONY, MDR-1000X). Before starting the experiment, an introductory message was displayed on the screen giving instructions for the experiment. The introductory message was presented in the subject’s native language, i.e., Japanese version for the JNS and Chinese version for the CLJ groups, to help the subjects better understand and apply it to the experiment. Participants could start the perception experiment with a single mouse click, and the first stimulus was automatically displayed. Within the trial, two alternatives were shown using *hiragana*, representing a singleton word and a geminate word (e.g., 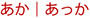). No *kanji* or *romaji* characters were used in the experimental options. The participants were instructed to choose which word they heard from the two options, and the next set was displayed immediately after they had made a choice. The experiment procedure was self-paced, and no time limit was set for the stimuli. Participants could also use two function buttons, “*Saisei* (replay)” and “*Modoru* (return),” to replay the current trial a maximum of three times or return to the previous question and change their answer, respectively.

For the test, 108 stimuli were divided into four blocks of 27. Each block was presented randomly to perceivers, and the stimulus order within each block was also randomized. A one-minute optional break was allowed at the end of every two blocks to avoid fatigue-related mistakes. Participants completed the identification task in approximately 30 min.

### Analyses and results for experiment 1

The data of perception experiment 1 were analyzed to measure the perceptual accuracies of Japanese singleton and geminate words. Perceptual accuracy was calculated for each perceiver, and we adopted the average value for each group of subjects.

The result of perceptual accuracy shows dispersion and a scattering of perceptual accuracy across a relatively wide range, except for the group of JNS. It is also found that the perceptual accuracy varies from singleton to geminate, especially for those in the Mandarin-speaking learner groups. Average percentages of correct responses for singleton and geminate words by five groups were calculated (see results in Table 2).

**TABLE 2:** Mean accuracies for words with singleton vs. geminate among the five groups.

Group	Average accuracy (%)	
	Singleton	Geminate
JNS	98.15 (*SE* = 0.55)	95.74 (*SE* = 1.13)
MSL-B	89.20 (*SE* = 1.70)	63.89 (*SE* = 2.54)
MSL-A	90.00 (*SE* = 1.58)	82.22 (*SE* = 2.48)
CSL-B	85.69 (*SE* = 1.84)	89.23 (*SE* = 1.66)
CSL-A	92.41 (*SE* = 1.32)	87.41 (*SE* = 1.73)

The data of perception experiment 1 was also analyzed to measure the perceptual sensitivity to take perceivers’ response bias into account (Macmillan and Creelman, 2005). Signal detection theory was adopted to conceive of perceptual sensitivity in detecting singleton/geminate contrasts. Thus, since perception experiment 1 was carried out under the condition of two-alternative forced-choice (2AFC) recognition, perceptual sensitivity, which is interpreted by *d*’ value, was computed by the formula as follows. By this means, the greatest possible sensitivity (using 1 as H and 0 as F) is 6.0811.

$$d^{\prime }=\frac{1}{\sqrt{2}}\,\left[z_{H}-z_{F}\right]$$

**FIGURE 1:**
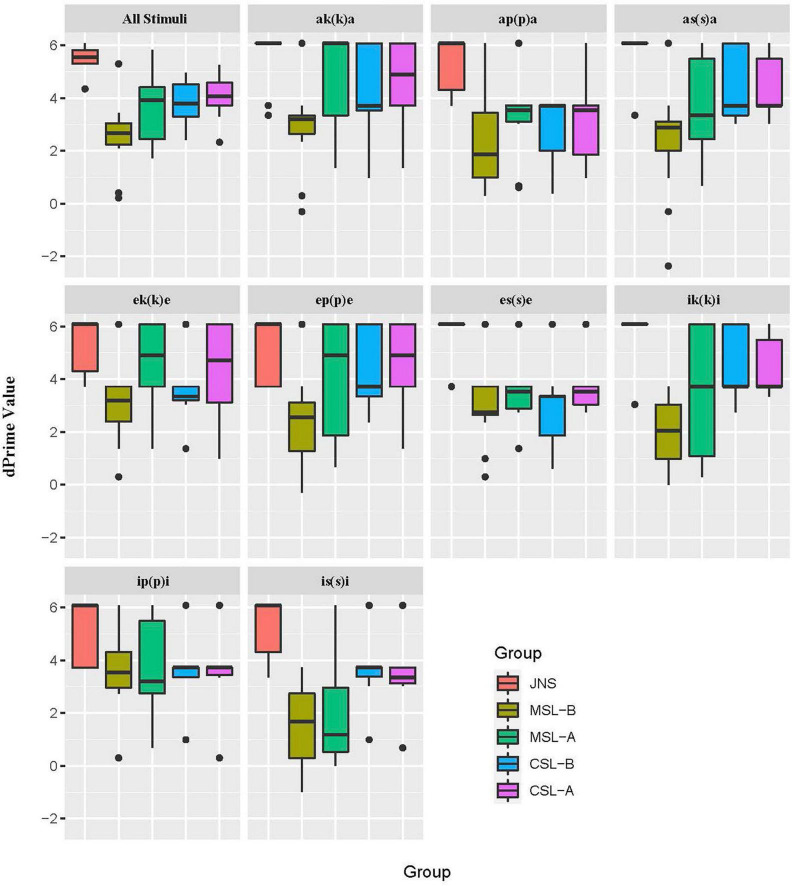
The average perceptual sensitivity by all groups of participants for all non-synthesized stimuli and for each minimal pair. –B, beginner group; –A, advanced group (e.g., MSL-B, beginner group of Mandarin-speaking learners; CSL-A, advanced Cantonese-speaking learner group).

Figure 1 displays the average perceptual sensitivity by all groups of participants. Differences in average dPrime value across groups are easily observed. That is, JNS group shows a particularly higher dPrime value than any other learner group, while MSL-B group shows the lowest perceptual sensitivity for Japanese singleton/geminate contrasts.

A one-way ANOVA was carried out in R version 4.2.0, examining the influence of the participant’s group (i.e., JNS, MSL-B, MSL-A, CSL-B, CSL-A) on dPrime values. The result showed that the four learner groups had a significantly lower perceptual sensitivity than the JNS (*p* < 0.001), regardless of learner’s L1 and Japanese proficiency. Also, the fact that the perceptual sensitivity of JNS is utterly independent of vowel and consonant types was also confirmed by a two-way ANOVA (*p*_*vowel*_ = 0.319, *n.s.*; *p*_*consonant*_ = 0.057, *n.s.*).

A four-way ANOVA was then conducted to fit the learners’ dPrime values. Independent variables of learners’ L1 (Mandarin, Cantonese), Japanese proficiency (beginner, advanced), surrounding vowel types (/a, e, i/), consonant types (/k, p, s/), as well as their interactions were included. The result of the ANOVA is summarized in Table 3.

**TABLE 3:** The ANOVA summary: ‘dPrime’ ∼ L1 * Japanese proficiency * vowel types * consonant types.

Factor level	df	*F*-value	Pr (>F)
L1 (Mandarin vs. Cantonese)	1	25.334	< 0.001***
Japanese proficiency (beginner vs. advanced)	1	16.035	< 0.001***
Vowel type (/a/ vs. /e/ vs. /i/)	2	1.528	0.218
Consonant type (/k/ vs. /p/ vs. /s/)	2	4.113	0.017*
L1: Japanese proficiency	1	5.848	0.016*
L1: Vowel type	2	1.188	0.306
L1: Consonant type	2	1.538	0.216
Japanese proficiency: Vowel type	2	1.051	0.351
Japanese proficiency: Consonant type	2	0.717	0.489
Vowel type: Consonant type	4	3.602	0.007**
L1: Japanese proficiency: Vowel type	2	0.426	0.654
L1: Japanese proficiency: Consonant type	2	0.736	0.480
L1: Vowel type: Consonant type	4	3.500	0.008**
Japanese proficiency: Vowel type: Consonant type	4	0.109	0.979
L1: Japanese proficiency: Vowel type: Consonant type	4	0.777	0.541

Significance codes: ***p < 0.001; **p < 0.01; *p < 0.05.

Results revealed significant main effects of L1 (*p* < 0.001) and Japanese proficiency (*p* < 0.001) on learners’ dPrime values. This demonstrated that CSLs showed significantly higher sensitivity in identifying Japanese singleton/geminate contrast than MSLs, and the overall sensitivity of learners’ perception improved significantly as the Japanese language proficiency increased. The main effect of surrounding consonant types was also significant (*p* < 0.05). Dunnett’s Modified Tukey–Kramer pairwise multiple comparison post hoc tests for the dPrime values with different consonant types were conducted. It was found that difference between /k/ and /s/ was significant (*p* < 0.05), showing that learners were less sensitive in perceiving contrasts with /s/ than those with /k/.

The interactions of L1 × Japanese proficiency (*p* < 0.05) and vowel × consonant type (*p* < 0.01) were shown to be significant, while a significant three-way interaction of L1, vowel type, and consonant type (*p* < 0.01) was also found to affect the perceptual sensitivity of Japanese singleton/geminate contrasts. Multiple comparisons of the L1 × Japanese proficiency interaction showed that the perceptual sensitivity of Japanese singleton/geminate contrasts for CSL was significantly higher than MSL when learners were at the beginner level (*p* < 0.001), while the difference between the advanced groups of MSL and CSL occurred but failed to reach significance (*p* = 0.225). Meanwhile, when we fixed the learners’ L1, a significantly higher perceptual sensitivity of singleton/geminate contrasts for advanced learners over beginning learners was observed in the MSL group (*p* < 0.001) but not in the CSL group (*p* = 0.510, *n.s.*). This may be evidence that the use of long and short consonants in learners’ L1 (Cantonese) blocks the effect of proficiency level. Multiple comparisons of the vowel × consonant interaction showed interesting results as well. Specifically, the effect of vowel type that achieved significance in this comparison was those examining perceptual sensitivity of /ap(p)a/ vs. /ep(p)e/ (β = –0.962, *p* < 0.05), as well as /as(s)a/ vs. /is(s)i/ (β = 0.986, *p* < 0.05). For single contrasts for consonant type, significant difference was also found in /ak(k)a/ vs. /ap(p)a/ (β = 1.188, *p* < 0.01) and /ip(p)i/ vs. /is(s)i/ (β = 0.907, *p* < 0.05). Breakdowns of the three-way interaction were adopted to examine how one, or more, two-way interactions differ across the levels of a third variable. The results achieved a significance level of 0.05 were summarized in Table 4.

**TABLE 4:** The three-way interaction (L1 × vowel × consonant) summary: The significant results in pairwise combination.

Fixed variables								
1		2		3rd variable		Estimated coefficient	*t* ratio	*P*-value
Vowel	/a/	Consonant	/s/	L1	MSL *vs.* CSL	–1.565	–3.021	0.003**
	/e/		/p/			–1.337	–2.580	0.010**
	/i/		/k/			–1.710	–3.302	0.001**
			/s/			–1.986	–3.834	< 0.001***
L1	MSL	Vowel	/i/	Consonant	/p/ *vs.* /s/	1.932	3.772	< 0.001***
		Consonant	/s/	Vowel	/e/ *vs.* /i/	1.486	2.902	0.011*
	CSL	Vowel	/a/	Consonant	/k/ *vs.* /p/	1.354	2.583	0.027*
					/p/ *vs.* /s/	–1.524	–2.908	0.011*
		Consonant	/p/	Vowel	/a/ *vs.* /e/	–1.509	–2.880	0.012*

Significance codes: ***p < 0.001; **p < 0.01; *p < 0.05.

## Perception experiment 2

### Methods

#### Participants

Same perceivers from perception experiment 1 participated in perception experiment 2 (see above). One female Japanese native speaker (Tokyo dialect), different from the female speaker who produced the stimuli for experiment 1, was asked to do the recordings. The speaker herself was a phonetician who was in her fifties.

#### Stimuli

Speech materials were recorded on the basis of the test word list described above. The recording was carried out in a sound-attenuated studio on the university campus in Tokyo. The speaker produced all the target words embedded in a carrier sentence “*Sore wa (test word) desu*.” Meaning “That is *(test word)*.” printed on paper in randomized order. The speaker was asked to slightly make a pause between the “*wa*” and the test word. A total of 108 (18 words × 6 times) short sentences were uttered by the speaker at her own natural speed. Since word accent is controlled as initial-accented pattern, all test words were produced in pitch accent type of HL (singleton) or HLL (geminate). The speech materials were recorded using a SONY F-780 microphone via a digital sound recorder (MarantzPMD 561). All recordings were digitized at 44.1 kHz with the quantization of 16-bit. A total of nine recordings (one for each minimal pair of singleton-geminate words such as /aka/ vs. /aQka/, /iki/ vs. /iQki/, etc.)^3^ were selected, and only the test word parts were excised from the recordings for sound synthesizing. The segment durations and CD/EWD values of each test word before synthesizing are presented in Supplementary Appendix 3.

To create a ten-step continuum for each minimal pair, stimuli were generated from the selected original recordings and were synthesized in Adobe Audition CC (version 10.1.1.11). We first manipulated the duration of the steady-state of preceding and following vowels to 100 and 150 ms^4^, respectively. The duration of preceding and following vowels in all stimuli was held constant after this manipulation. Then, CD/EWD was shortened or lengthened in 10 steps by manipulating the central portion of the constriction duration in each stimulus. The range and step size of the stimuli continuum were from 15 to 60% in 5% increments, depending on the results of a pilot experiment. The experiment 2 also invoked the segmentation criteria used in Fischer-Jørgensen and Hutters (1981). All synthesized sounds were normalized to 65 dB, and 500 ms of white noise was added before and after all synthesized sounds. At this point, syntheses of stimuli were completed, and a total of 90 (9 minimal pairs × 10 steps) were created to adopt to the categorial identification test (CIT) of Japanese singleton and geminate contrast.

#### Procedure

Perceivers were tested in a quiet room at universities in Japan or China, seated alone at a table in front of a computer (without the experimenter). Stimuli were presented in isolation (e.g., /ap(p)a/, /ik(k)i/, /es(s)e/, etc.) to participants using the ExperimentMFC function of Praat (version 6.0.34). All stimuli were displayed at a self-selected comfortable volume through wireless circumaural headphone (SONY, MDR-1000X). Upon perceiving each stimulus, two possible responses in hiragana, such as ‘
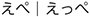
’ were shown on the monitor. Participants were asked to make a two-alternative forced-choice between a singleton or geminate word. “*Saisei* (replay)” and “*Modoru* (return)” buttons were also displayed, making it possible to replay the current question and return to the previous one, respectively.

All stimuli were presented to perceivers twice in random order, yielding a total of 180 questions in this section. This categorical identification test was broken into two sessions (90 stimuli for each session). This could help the perceivers to take a break and concentrate on every question. Each testing session contained three blocks, and each block had 30 stimuli. The blocks and stimuli were formed randomly. The experimental session was self-paced, and participants took approximately 45 min to complete the experiment. This experiment was conducted on separate days from experiment 1.

### Analyses and results for experiment 2

#### Average perceptual identification percentage

**FIGURE 2:**
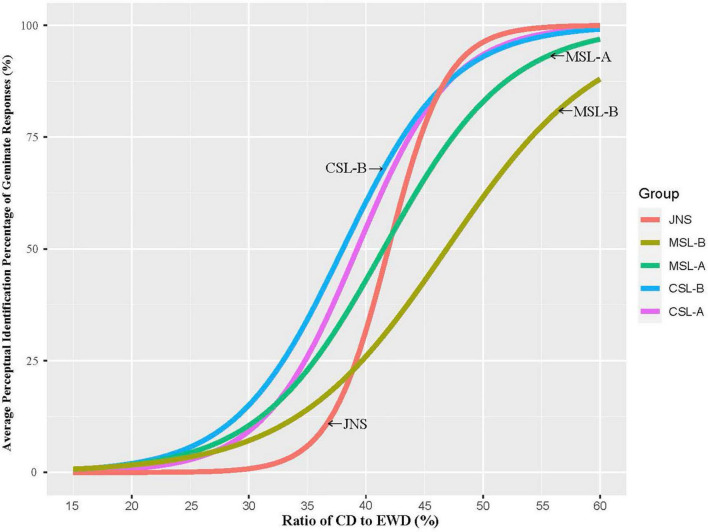
Approximate curves of logistic regression models to the average percentage of geminate (‘S’) responses.

To understand the categorical perception of Japanese singleton and geminate contrasts, the perceptual identification percentage for stimuli with different CD/EWD was calculated by groups. Approximate curves based on fits of logistic regression models to the average proportion of geminate (‘S’) responses by group are shown in Figure 2. As illustrated in Figure 2, responses for each group showed that the perceived percentage of Japanese geminates jumped abruptly from one to another and the identification data appeared to have steep slopes at category boundaries. The logistic function for each group was tested for significance using likelihood ratio tests (*p* < 0.001). This suggested strong evidence that Japanese singletons and geminate were perceived categorically with a clear boundary point of CD/EWD by participants from all groups.

#### Slope of perception curve and categorical boundary point

**FIGURE 3:**
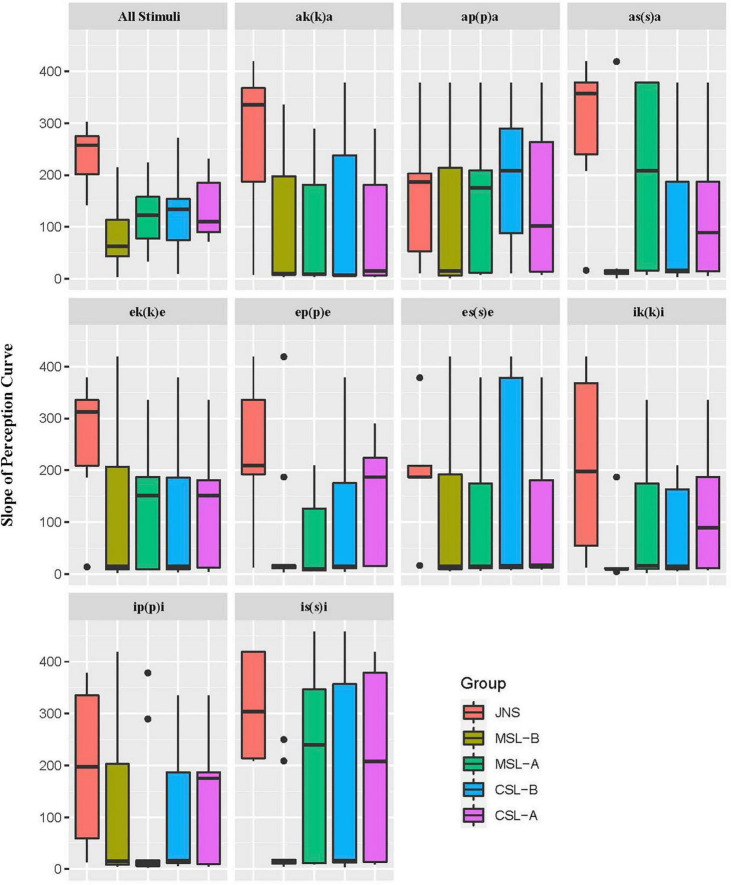
Average values of slope of perception curve by five groups for all stimuli and for each minimal pair.

**FIGURE 4:**
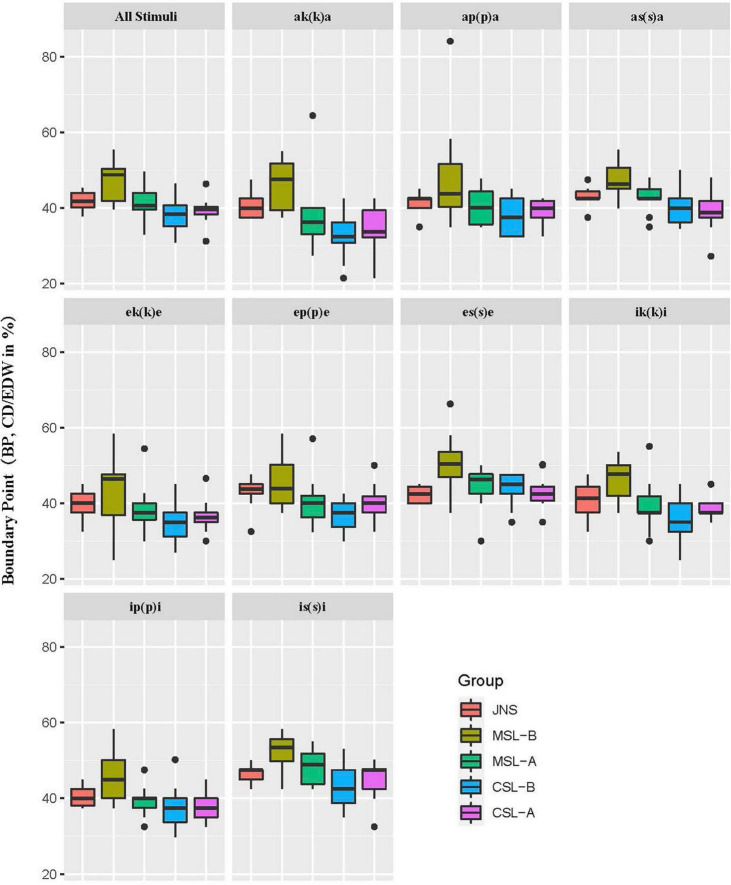
Average values of categorical boundary point by five groups for all stimuli and for each minimal pair.

When examining categorical perception, the slope of the perception curve and the categorical boundary point (BP) are two parameters often used. By adopting these two parameters, we can see whether subjects identify Japanese singleton/geminate contrasts categorically by adopting CD/EWD as a cue. Logistic regression was used to model the categorical perception. Model fitting was carried out in R version 4.2.0 using the lme4 package (Bates et al., 2015). The slope and the categorical boundary point were defined by slope coefficients and the CD/EWD value at the point that gave a 50% proportion of geminate response derived from logistic regression models, respectively. The overall results of both slope and boundary point of Japanese singleton and geminate contrasts by each group are illustrated in Figures 3, 4, respectively.

A one-way MANOVA was conducted using R version 4.2.0 to examine differences in average categorical slope and BP values (the dependent variables) by different groups of perceivers (independent variable). The results showed that JNS elicited significantly larger slope values than any other group of learners (*p* < 0.001). The combination of the BP values, however, exhibited interesting results. First, the MSL-B group demonstrated significant differences from the other learner groups. Specifically, the boundary for the MSL-B group is larger than that for the native perceivers, while the other three groups are lower than the JNS group. Second, the difference in boundary point between the JNS group and the MSL-A group was insignificant (*p* = 0.980, *n.s.*). In contrast, the BPs for the others were significantly different from that of the JNS (MSL-B and CSL-B: *p* < 0.001; CSL-A: *p* < 0.05).

Meanwhile, a two-way MANOVA was conducted to test whether vowel type, consonant type and their interaction affected the categorical slope and BP values of JNS. The results revealed that the vowel type did not have any significant effect on slope (*p* = 0.754, *n.s.*) and BP (*p* = 0.076, *n.s.*). In contrast, consonant type and its interaction with vowel type only had a significant effect on BP values (*p* < 0.05). By pairwise comparison for this interaction of vowel type × consonant type, we found that the BP value of /is(s)i/ was significantly larger than that of /ik(k)i/ and /ip(p)i/ (*p* < 0.001) when the vowel type was fixed to /i/. Also, when the consonant type was fixed to /s/, the BP value of /is(s)i/ was significantly larger than /as(s)a/ and /es(s)e/ (*p* < 0.001). It is clear from the above tendencies that all these significant differences were derived from /is(s)i/. Therefore, we may conclude that the seemingly significant main effect of consonant type on BP values is actually due to the palatalization caused by vowel /i/ with the preceding consonant /s/. Combining the results in Experiment 1, the possibility that vowel and consonant type affect the categorical perception of Japanese singleton/geminate contrasts by JNS might be basically excluded.

For the analysis of the categorical perception of Japanese singleton/geminate contrasts for Chinese learners of Japanese, statistical analysis using MANOVA function was then carried out again. Four factors (learner’s L1, Japanese proficiency, surrounding vowel and consonant types of the stimuli) were adopted as independent variables, while the slope and BP values as dependent variables, respectively. The results of the MANOVA are summarized in Table 5.

**TABLE 5:** The MANOVA summary: ‘Slope + boundary point’ ∼ L1 * Japanese proficiency * vowel types * consonant types.

Factor level	Slope			Boundary point		
	df	*F*-value	Pr (>F)	df	*F*-value	Pr (>F)
L1 (Mandarin vs. Cantonese)	1	4.772	0.030*	1	89.333	< 0.001***
Japanese proficiency (beginner vs. advanced)	1	3.057	0.081	1	14.416	< 0.001***
Vowel type (/a/ vs. /e/ vs. /i/)	2	0.264	0.768	2	2.631	0.073
Consonant type (/k/ vs. /p/ vs. /s/)	2	1.314	0.270	2	32.772	< 0.001***
L1: Japanese proficiency	1	1.846	0.175	1	31.434	< 0.001***
L1: Vowel type	2	0.244	0.784	2	0.932	0.395
L1: Consonant type	2	0.275	0.760	2	0.482	0.618
Japanese proficiency: Vowel type	2	0.923	0.398	2	0.370	0.691
Japanese proficiency: Consonant type	2	1036	0.356	2	0.033	0.967
Vowel type: Consonant type	4	2.268	0.062	4	2.949	0.020*
L1: Japanese proficiency: Vowel type	2	1.005	0.367	2	0.058	0.944
L1: Japanese proficiency: Consonant type	2	1.155	0.316	2	0.854	0.427
L1: Vowel type: Consonant type	4	0.342	0.850	4	0.362	0.836
Japanese proficiency: Vowel type: Consonant type	4	1.477	0.209	4	0.917	0.866
L1: Japanese proficiency: Vowel type: Consonant type	4	0.173	0.952	4	0.200	0.938

Significance codes: ***p < 0.001; **p < 0.01; *p < 0.05.

The summarized MANOVA showed that the predictor of subjects’ L1 (*p* < 0.05) was significant on the slope of the perception curve. Only learners’ L1 was confirmed to significantly affect the sharpness of categorical perception of Japanese singleton/geminate contrasts, showing that CSL is significantly higher than MSL. At the same time, other variables, as well as the interactions, failed to reach significance. This result also implies having a clearer and more distinct categorical perception of CSL than MSL. The Japanese proficiency, surrounding vowel/medial consonant type as well as all interactions between factors yielded no significant influence on the slope values.

The results for the boundary point showed that three main effects other than the surrounding vowel type were significant at the 0.001 level. Also, the interaction of L1 × Japanese proficiency (*p* < 0.001) as well as vowel × consonant (*p* < 0.05) was shown to be significant. Multiple pairwise comparisons were performed to establish which are the significant differences. The results firstly confirmed that the MSL showed a significantly larger BP value than CSL. Combined with the results of JNS, there is an overall tendency of CSL < JNS < MSL in terms of BP values, and a significant difference at 0.01 level was seen between the three groups. As for the main effect of Japanese proficiency, differences in BP value between the beginner and advanced learner groups were also significant, demonstrating significantly larger BP for beginning learners while significantly smaller BP for advanced learners, compared to the JNS. It also revealed that the boundary point of contrasts with consonant /s/ was significantly larger than those with /k/ and /p/ (*p* < 0.001), while the difference between the two plosives was likewise significant (/p/ > /k/, *p* < 0.05). This indicates that perceivers need more constriction duration period when distinguishing singleton and geminate contrasts with fricatives, compared with those containing plosives. Moreover, the results of multiple pairwise comparisons also suggest an interesting joint effect of L1 across learners’ Japanese proficiency on the categorical boundary point. That is, learners’ L1 had a significant impact on BP values when learners were at both the beginner (*p* < 0.001) and advanced (*p* < 0.05) stages. Yet, the difference in learners’ Japanese proficiency arose significant difference in the BP values only in the MSL group (*p* < 0.001). The interaction between vowel type and consonant type was also significant. Specifically, when concerning simple contrasts for vowel, the vowel /a/ demonstrated a lower BP than /i/ (*p* < 0.001) when the consonant is fixed to /s/. Also, when concerning simple contrasts for consonant, we observed a tendency that the average value of BP for words with /k/ was lower than /s/ in all three vowel conditions (*p* < 0.001). Thus, the consonant /p/ demonstrated a lower BP than /s/ (*p* < 0.001) when the vowels are /e/ (*p* < 0.05) and /i/ (*p* < 0.001).

## Discussion

The present study investigated the perception of Japanese singleton and geminate contrasts by JNS and learners of Japanese who come from different dialectal backgrounds. Two separate experiments, in which both natural sounds and synthesized sounds were adopted, were conducted to solve the questions addressed in the Introduction.

Of primary concern in the present study is how learners of Japanese perceive singleton and geminate contrasts in comparison with JNS. Experiment 1 tested the average perceptual accuracy of singletons and geminates. The results revealed that JNS perceives singletons and geminates at a high level without distinction. In contrast, perceptual accuracy between singletons and geminates was different for learners of Japanese, especially in the case of Mandarin-speaking learners. Considering that the combination with separate perceptual accuracy scores of singletons and geminates has limitations due to the response bias, d’- based analyses were also performed to clarify the perceptual sensitivity in detecting singleton/geminate contrasts for both Japanese native speakers and Chinese learners of Japanese. Overall, perceptual performance in experiment 1 for JNS group was best when identifying Japanese singletons and geminates, followed by the two Chinese learner groups, with significant differences. The result revealed that learners of Japanese could not reach the same high level as JNS in perceiving Japanese singletons and geminates, regardless of their dialectal backgrounds. Consistent findings have also been confirmed in experiment 2, showing that there is a certain gap in the results of categorical perception (i.e., slope and BP value) for Japanese singleton/geminate contrasts between JNS and CLJ, despite the diversity in L1 and Japanese proficiency across all learner groups. Taken together, the results of the present investigation generally support the idea that perceiving singleton and geminate contrasts accurately is particularly problematic in the acquisition of L2 speech for learners of Japanese (Nishibata, 1993; Uchida, 1993; Toda, 1998; Jia et al., 2005; Kondo, 2011).

The present study also analyzed the extent to which the Chinese learner groups differed from each other with regard to the perceptual sensitivity and categorical perception of Japanese singleton and geminate contrasts. As for perceptual sensitivity of naturally pronounced singleton and geminate contrasts, Mandarin-speaking learners who have neither checked tones nor long consonant sequences in their dialect were shown to be less accurate than Cantonese-speaking learners at the beginner stage when perceiving Japanese geminates, as predicted. These findings could be interpreted as evidence to support McAllister et al. (2002) that challenges in obtaining an L2 contrastive category are associated to some extent with the role in the L1 of the phonetic characteristic, which refers to the L2 category. Specifically, the presence of long consonant sequences in Cantonese, which are generally manifested as checked tones, could positively affect the process of CSL to perform better at identifying Japanese singletons and geminates compared to MSL with the assistance of the similar phonetic status in their L1 phonology. Similar to the results of experiment 1, the finding that learners with different dialectal backgrounds differ greatly in the slope of categorical perception provides further evidence that positive impact motivated by learners’ L1 indeed occurred in the case of Cantonese-speaking learners due to phonetic similarities (i.e., long consonant sequences). A better performance, possibly due to positive transfer resulting from the L1 of CSL, was also observed in the BP (in CD/EWD proportion) of categorical perception when we compared the distance to JNS on BP between beginner groups of MSL and CSL^5^. Yet, when groups with different Japanese proficiencies are taken together, MSL showed a more approximate boundary point to JNS in comparison with CSL. This was only because the advanced MSL group used a BP in categorizing Japanese singletons and geminates without significant differences with that of JNS. It is worth pointing out the possibility that the learner’s developmental progress could alleviate the difficulties in the acquisition of L2 phonology caused by the unexploited features in the learner’s L1. Also, from the results of the BP in categorical perception, CSL groups seem to be consistently showing lower categorical boundaries than native speakers, while MSL groups performed very far from native speakers as the beginning learners, but got very close to the native speakers’ value for the advanced group (see Figure 2). This may actually show a different kind of challenge for CSL groups, which is expressed as “new wine in old bottles” in Flege (1998) – because they have some equivalents similar to Japanese singleton/geminate consonants in Cantonese, they have a head start initially but have a hard time adjusting more precisely to the native-model boundary values (i.e., consistently lower than JNS in BP).

The findings of the current study also suggested acoustic cues for the perception of Japanese singleton and geminate contrasts from robust durational cues, such as constriction duration, as suggested in previous studies. In our analysis, it should be noted that the surrounding acoustic environment did influence the perception of Japanese singleton and geminate contrasts regardless of learners’ language background or Japanese proficiency. We used test words with three different types of vowels (high, mid, low) as stimuli. The results of the present investigation indicated that preceding and following vowels seem to have little effect on the perception of Japanese gemination, besides the interaction of vowel and consonant types on categorical BP. However, Fujimoto and Maekawa (2014) found that the effect of Japanese geminates on the preceding vowel is robust in both stop consonants and fricatives acoustically. In other words, the preceding vowel should be considered a distinct correlate in distinguishing between singletons and geminates. Additionally, the perception of Japanese geminates significantly differs when the length of the preceding and following vowels varies (Ofuka et al., 2005). This strongly suggests that vowels, to a certain extent, have some effect on the perception of Japanese geminates. Taken together, results from previous studies provide interesting possibilities to re-examine the impact of vowels on perception performances by modifying the duration of surrounding vowels or using all five vowels in the Japanese language. On the other hand, as a secondary cue, the medial consonant type would seem to affect learners in distinguishing Japanese singleton and geminate contrasts more than vowel type. More specifically, singleton and geminate contrasts with a medial consonant /p/ were predicted to be less perceptible since the durational difference between /p/ and /pp/ (i.e., the ratio of geminate duration to singleton duration) is reported to be the slightest among all plosives (Homma, 1981). The perception sensitivity results by Chinese Japanese learners seemed to be inconsistent with our prediction. It showed the second highest perception sensitivity among the three medial consonant types for contrasts with plosive /p/, with no significant difference between contrasts with /k/ (*p* = 0.180, *n.s.*), which showed the highest. A possible explanation might be the sonority differences between the target consonant and the neighboring vowels, as Hardison and Motohashi-Saigo (2010) suggested. Specifically, the sonority difference between the consonant and following vowel is positively correlated with the strength of a segment’s cues in terms of perception (Wright, 2004). As in Selkirk’s (1984) sonority index, plosives /p/ and /k/ are given the same value of 0.5, fricative /s/ is 4, and the vowels are from 8 to 10. The sonority difference is therefore greater for a plosive + vowel sequence (e.g., /epe/) than a fricative + vowel sequence (e.g., /ese/), representing the same higher perceptual sensitivity for contrasts with plosives compared with contexts with fricatives.

It is also worth pointing out that the fricative /s/ appears to have a more distinct feature in terms of perceptual sensitivity and categorical perception of Japanese singleton/geminate contrasts, in contrast with plosives. In the current study, learners of Japanese showed the least sensitivity in identifying singleton and geminate contrasts with /s/. They also tended to need a longer duration (CD/EWD) to distinguish between singletons and geminates with medial consonant /s/, indicating that it is more challenging for learners to categorize /s/ vs. /ss/. The fact that plosives and fricatives are pronounced differently in gemination^6^ indeed may lead to different perception tendencies. In lengthened plosives, the obstruction of the airway is prolonged, which delays release, and the “hold” is lengthened. This sense of “hold” is considered an important hint in the perception of Japanese geminate consonants. However, when fricative /s/ becomes a long-duration version in gemination, there is no sense of oral stop closure as in plosives and no change in sound quality. This suggests that the fricative portion of a geminate possesses the same status as a simple fricative, except for the difference only in duration, and could inevitably result in poor discrimination between /s/ and /ss/. The duration of Japanese singleton fricatives is reported to be longer than singleton stops in general (e.g., Beckman, 1982; Sagisaka and Tohkura, 1984; Campbell, 1999), which could be interpreted as smaller duration ratios of geminates to singletons for fricatives than for stops, as noted in Kawahara (2015). Smaller differences in duration between singletons and geminates cause less distinct discrimination and this can be considered another possible reason for the difficulty in the perception of Japanese gemination with /s/ as the medial consonant. Thus, Japanese vowel /i/ causes palatalization of the preceding consonant /s/, meaning that test words /isi/ are phonetically [iɕi]. This makes it inappropriate to make simple comparisons among the three consonants. Accordingly, MANOVA was conducted to compare whether there are significant differences between test words /as(s)a/, /es(s)e/ and /is(s)i/, by adopting dPrime value, categorical perception slope and BP value as dependent variables, as well as the three test word types as independent variable. Results for both dPrime and BP values were found significant. Afterwards, by conducting post hoc test, we found that those significant differences were derived from the test words /as(s)a/ and /is(s)i/, indicating more sensitive perception for words with vowel /a/ than /i/ (*p* < 0.05), and larger categorical BP for /is(s)i/ than /as(s)a/ (*p* < 0.01). Therefore, the possibility that palatalization caused by vowel /i/ may somehow influence the perception of Japanese singleton/geminate contrasts should be considered, eliciting the necessity for further investigation.

The categorical perception experiment corroborates previous research related to relational timing cues for discriminating Japanese singletons and geminates. It has been claimed that duration ratios like C/Mora_1_ and C/V_2_ could indeed be used as perceptual cues (Idemaru and Guion, 2010). The present study expands relational timing factors by varying CD/EWD to examine its function in the perception of Japanese singletons and geminates. Results from the current study demonstrated that CD/EWD is a reliable durational cue in discriminating Japanese singletons and geminates. In Ren (2017), *a priori* study, we explored the perception of geminate consonants by Chinese learners of Japanese. The raw closure duration in /aka/, /apa/, and /ata/ were scaled to 60∼250 ms with a 10-ms step size, and a total of 60 stimuli were used in the research. The results showed that the slope of the categorization function of Japanese singleton/geminate contrasts with raw closure duration as the cue was gradient, especially for beginning learners, who showed category perception regarding low-status stability in a relatively wide range of closure duration from 100 to 200 ms. Similarly, the results for BP among both beginner and advanced learners were far from those of Japanese native speakers, showing considerable variation within each group. In contrast with the results from Ren (2017), in which participants adopted raw constriction duration as the perceptual cue, learners’ performance on categorical perception of Japanese gemination was much more stable using CD/EWD as a relational timing cue. It also showed more explicit BP between singleton and geminate categories relying on CD/EWD as a variable, without great variability. Moreover, in the present study, we obtained stimulus sequences with varying CD/EWD ratios by manipulating only the test words’ constriction duration while keeping the preceding and following vowels constant. Although such kind of CD/EWD ratio can be seen as a particular form of relational timing since it is proportionally processed, the entire word duration should be simply a reflection of this constriction duration manipulation. It is conceivable that the listeners could have performed the task by focusing on constriction duration-related cues other than CD/EWD. Hence, it is worthwhile to further investigate whether different perceptual tendencies would be observed in the stimuli with various CD/EWD by varying both the surrounding vowels and constriction duration. This could also help to further clarify the reliability of CD/EWD as a relative duration cue.

With regard to other reliable cues on the perception of Japanese gemination by learners of Japanese, this study examined to what extent the Japanese proficiency of learners affects the perception of Japanese singleton and geminate contrasts. Results of statistical analyses showed the main effects of Japanese proficiency on not only the perception sensitivity of naturally pronounced singletons and geminates but also the categorical perception of those contrasts. However, when taking learners’ dialects into account, we could easily recognize that the perception of Mandarin-speaking learners varies greatly with the level of Japanese proficiency. Specifically, it shows a perceptual tendency to be more accurate or closer to the level of JNS as their language proficiency improves. On the other hand, for CSL groups, significant developmental progress was only observed in the perceptual sensitivity, which seems to have increased from the beginning to the advanced. Nonetheless, in a very specific sense of categorical boundaries, the advanced Cantonese group’s boundary point was still not identical to the native speakers’. From the results of Experiment 2, we can say that slope values of CSL are greater than those of MSL while BP values of CSL are smaller than those of MSL (although BP values of CSL do not perfectly match those of JNS). This means that CSLs are more categorical in singleton/geminate judgments than MSLs, and that CSLs have less response bias for singletons than MSLs. The fact that Cantonese has false geminates and CSLs can more categorically identify singletons/geminates than MSLs would also be evidence for positive transfer. In addition, as mentioned above, it is clear that Japanese proficiency interacts with learners’ dialectal background in the perception of Japanese singletons and geminates. Learners of Japanese with L1 dialects in which certain phonetic items can stimulate positive transfer in perceiving singleton and geminate contrasts are less likely to be affected by Japanese proficiency. For other learners, higher Japanese language ability correlates with better performance in perceiving Japanese singleton and geminate contrasts.

This study provided some insight into the perceptual differences between JNS and CLJ on singleton and geminate contrasts and cues that affect perception. The findings may have direct implications for Chinese learners to improve their perception of L2 Japanese. Meanwhile, the results of the present study provide evidence for cross-linguistic impact on the perception of Japanese gemination, which should be considered that learners from different L1 backgrounds should be trained according to their aptitude to achieve a better acquisition. It has to be mentioned that one of the limitations of the current study is the limited sample size, as well as the range of Chinese dialects (learner’s L1), due to the excluded subjects when strict criteria were used to recruit appropriate participants. To some extent, this may have led to the inability of the study to draw more general conclusions about the impact of learners’ L1 and Japanese proficiency on the perception of Japanese geminate consonants. However, the overall number of utterances in each experiment is relatively large and sufficient, which is the reason for the good credibility of the study. Thus, the findings of the current study still have important implications for clarifying learners’ differential categorical perception patterns in L2 Japanese. As several Chinese southern dialects have checked tones, such as Hakka, Min, and Wu, it would be interesting to replicate the experiments in future studies with a relatively more significant number of participants with a more comprehensive range of Chinese dialects.

## Notes

(1) Both real words and none words were used as test words which would possibly affect the perceptual outcomes. However, it will be difficult to maintain the consistency of the stimuli in terms of whether they express meanings or not while keeping stimuli with the same surroundings (vowel and medial consonant types) and pitch accent pattern.

(2) Hirata and Lambacher (2004) suggests that native speakers’ perception depends significantly on the speaking rate of the surrounding context, and perceptual judgments shift when a word is excised out of a sentential context. Since the methodology of excised stimuli perception was used in this study, slightly different results may be obtained if they were done in the original sentence context.

(3) Recordings were initially planned to be selected from both singleton and geminate words in the same proportion for synthesizing (e.g., four from singleton and five from geminate). However, we found it more accessible to shorten or lengthen the constriction duration of geminate words, and this can also avoid the perceptual differences due to synthesizing from words with different mora. Therefore, only geminate tokens were selected to create synthesized stimuli used in experiment 2.

(4) The duration of the preceding and following vowels in synthesized sounds was determined by rounding the average values (102, 153 ms) in all collected recordings to the nearest ten.

(5) The overall mean BP values (in CD/EWD proportion) were 41.91 for the JNS, 44.61 for the MSL, and 38.62 for the CSL. The absolute values of the difference in BP between the two learner groups (MSL and CSL) and the JNS were 2.7 and 3.29, respectively.

(6) Typically, if the consonant of the following mora in Japanese gemination is a plosive, then constriction of the first half of the plosive is held, forming a long consonant together with the following one. Corresponding to the case of plosives, if the following consonant in gemination is a fricative, a long-duration fricative is formed along with the following consonant.

## Data availability statement

The original contributions presented in this study are included in the article/Supplementary material, further inquiries can be directed to the corresponding author.

## Ethics statement

The studies involving human participants were reviewed and approved by Academic Research Ethical Review Committee of Waseda University. Written informed consent to participate in this study was provided by each participant.

## Author contributions

HR contributed to conception and design of the study, organized the database, performed the statistical analysis, and wrote the first draft of the manuscript.
