# Supplementary material for: Perception of Japanese singleton and geminate contrasts: A case of Chinese learners with different dialectal backgrounds
Source: Front Psychol. 2022 Dec 7;13:1070107. doi: 10.3389/fpsyg.2022.1070107 (PMC9768474; doi:10.3389/fpsyg.2022.1070107)
Supplement: Supplementary file 1 [file Data_Sheet_1.docx]

**Appendix I.** List of test words: nine minimal pairs with two syllables, in which one contains a geminate and another a singleton are used as test words in the perceptual experiments.

|  | /a/ | /e/ | /i/ |
| --- | --- | --- | --- |
| /k/ | /a(k)ka/ | /e(k)ke/ | /i(k)ki/ |
| /p/ | /a(p)pa/ | /e(p)pe/ | /i(p)pi/ |
| /s/ | /a(s)sa/ | /e(s)se/ | /i(s)si/ |

**Appendix** **Ⅱ.** The segment durations and CD/EWD values of each stimulus used in experiment 1.

| Sound Provider | Stimuli | Segment Durations (msec.) | | | EWD  (msec.) | CD/EWD  (%) |
| --- | --- | --- | --- | --- | --- | --- |
|  |  | Preceding Vowel Duration | Constriction Duration | Following Vowel Duration |  |  |
| SP-1 (Female) | /aka/ | 100.11 | 88.36 | 135.60 | 345.49 | 25.58 |
|  |  | 120.10 | 91.97 | 146.14 | 378.13 | 24.32 |
|  |  | 105.03 | 96.03 | 154.30 | 371.15 | 25.87 |
|  | /akka/ | 90.64 | 437.09 | 94.30 | 634.55 | 68.89 |
|  |  | 102.10 | 479.93 | 96.37 | 694.60 | 69.09 |
|  |  | 108.27 | 410.20 | 92.38 | 626.74 | 65.45 |
|  | /apa/ | 111.78 | 116.65 | 127.13 | 362.79 | 32.15 |
|  |  | 144.58 | 147.57 | 100.11 | 404.41 | 36.49 |
|  |  | 117.91 | 109.53 | 136.98 | 372.22 | 29.43 |
|  | /appa/ | 109.54 | 444.17 | 128.29 | 688.35 | 64.53 |
|  |  | 84.56 | 354.37 | 100.38 | 547.40 | 64.74 |
|  |  | 128.53 | 392.28 | 85.34 | 611.74 | 64.13 |
|  | /asa/ | 102.78 | 104.20 | 136.00 | 342.97 | 30.38 |
|  |  | 112.68 | 139.99 | 147.30 | 399.96 | 35.00 |
|  |  | 128.78 | 121.05 | 139.41 | 389.24 | 31.10 |
|  | /assa/ | 117.38 | 299.54 | 78.96 | 495.88 | 60.41 |
|  |  | 144.92 | 341.49 | 102.30 | 588.70 | 58.01 |
|  |  | 129.82 | 349.23 | 97.09 | 576.14 | 60.62 |
|  | /eke/ | 116.28 | 81.70 | 167.33 | 389.24 | 20.99 |
|  |  | 122.45 | 81.50 | 128.14 | 355.08 | 22.95 |
|  |  | 145.14 | 98.25 | 141.66 | 411.88 | 23.85 |
|  | /ekke/ | 124.40 | 316.75 | 104.66 | 560.12 | 56.55 |
|  |  | 126.84 | 396.97 | 134.99 | 679.01 | 58.46 |
|  |  | 137.40 | 465.05 | 130.37 | 759.36 | 61.24 |
|  | /epe/ | 120.50 | 137.16 | 124.98 | 391.79 | 35.01 |
|  |  | 132.76 | 115.77 | 153.65 | 407.73 | 28.39 |
|  |  | 99.41 | 118.62 | 99.69 | 324.49 | 36.56 |
|  | /eppe/ | 111.30 | 451.41 | 84.99 | 653.27 | 69.10 |
|  |  | 135.20 | 459.70 | 134.88 | 735.71 | 62.48 |
|  |  | 112.96 | 408.55 | 95.26 | 624.54 | 65.42 |
|  | /ese/ | 125.83 | 119.59 | 111.11 | 356.53 | 33.54 |
|  |  | 145.77 | 149.04 | 119.87 | 414.68 | 35.94 |
|  |  | 124.33 | 152.84 | 139.27 | 416.44 | 36.70 |
|  | /esse/ | 124.67 | 346.15 | 102.27 | 573.09 | 60.40 |
|  |  | 154.89 | 353.18 | 121.35 | 629.42 | 56.11 |
|  |  | 141.13 | 423.17 | 115.48 | 679.78 | 62.25 |
|  | /iki/ | 92.87 | 84.85 | 140.34 | 347.48 | 24.42 |
|  |  | 113.19 | 112.58 | 164.66 | 442.94 | 25.42 |
|  |  | 123.49 | 110.67 | 152.99 | 428.93 | 25.80 |
|  | /ikki/ | 104.36 | 419.77 | 85.13 | 674.98 | 62.19 |
|  |  | 120.44 | 473.39 | 88.30 | 722.47 | 65.52 |
|  |  | 125.53 | 423.53 | 99.80 | 688.71 | 61.50 |
|  | /ipi/ | 133.78 | 126.74 | 137.47 | 405.61 | 31.25 |
|  |  | 143.21 | 150.25 | 128.89 | 433.78 | 34.64 |
|  |  | 126.45 | 117.56 | 132.48 | 384.58 | 30.57 |
|  | /ippi/ | 130.59 | 332.50 | 118.41 | 588.66 | 56.48 |
|  |  | 138.05 | 381.09 | 108.18 | 634.38 | 60.07 |
|  |  | 130.51 | 445.22 | 108.83 | 691.73 | 64.36 |
|  | /isi/ | 117.35 | 172.62 | 117.41 | 407.38 | 42.37 |
|  |  | 109.26 | 194.87 | 91.50 | 395.62 | 49.26 |
|  |  | 123.88 | 185.45 | 97.83 | 407.16 | 45.55 |
|  | /issi/ | 133.65 | 365.42 | 97.08 | 596.15 | 61.30 |
|  |  | 132.90 | 388.55 | 104.99 | 626.44 | 62.03 |
|  |  | 130.26 | 411.17 | 95.52 | 636.96 | 64.55 |
| SP-2 (Male) | /aka/ | 90.59 | 91.51 | 112.65 | 320.86 | 28.52 |
|  |  | 108.36 | 85.95 | 113.19 | 331.10 | 25.96 |
|  |  | 103.63 | 88.61 | 118.83 | 336.35 | 26.34 |
|  | /akka/ | 99.47 | 209.01 | 108.62 | 444.94 | 46.97 |
|  |  | 105.16 | 240.90 | 105.70 | 479.58 | 50.23 |
|  |  | 94.60 | 204.06 | 113.93 | 438.65 | 46.52 |
|  | /apa/ | 74.16 | 126.45 | 107.76 | 316.24 | 39.99 |
|  |  | 99.00 | 107.42 | 123.11 | 337.26 | 31.85 |
|  |  | 97.76 | 131.34 | 110.03 | 347.64 | 37.78 |
|  | /appa/ | 99.45 | 264.87 | 122.03 | 496.44 | 53.35 |
|  |  | 73.94 | 243.18 | 88.38 | 414.08 | 58.73 |
|  |  | 108.89 | 235.08 | 95.85 | 449.13 | 52.34 |
|  | /asa/ | 119.25 | 92.05 | 145.25 | 356.54 | 25.82 |
|  |  | 110.13 | 112.58 | 123.84 | 346.54 | 32.49 |
|  |  | 102.54 | 119.41 | 139.33 | 361.28 | 33.05 |
|  | /assa/ | 106.40 | 242.75 | 141.97 | 491.12 | 49.43 |
|  |  | 101.42 | 283.21 | 110.87 | 495.50 | 57.16 |
|  |  | 110.00 | 238.59 | 100.35 | 448.95 | 53.14 |
|  | /eke/ | 96.70 | 88.79 | 133.64 | 341.81 | 25.98 |
|  |  | 96.74 | 91.00 | 130.58 | 343.30 | 26.51 |
|  |  | 73.67 | 72.69 | 127.30 | 294.32 | 24.70 |
|  | /ekke/ | 93.52 | 229.47 | 105.57 | 450.41 | 50.95 |
|  |  | 84.25 | 224.40 | 137.10 | 480.52 | 46.70 |
|  |  | 99.86 | 215.44 | 86.20 | 418.54 | 51.47 |
|  | /epe/ | 94.46 | 125.63 | 121.23 | 349.11 | 35.99 |
|  |  | 106.09 | 120.05 | 114.75 | 347.74 | 34.52 |
|  |  | 100.70 | 110.06 | 124.49 | 344.11 | 31.98 |
|  | /eppe/ | 82.59 | 242.67 | 83.12 | 417.14 | 58.17 |
|  |  | 95.94 | 251.29 | 99.91 | 456.27 | 55.07 |
|  |  | 69.86 | 252.88 | 80.60 | 412.17 | 61.35 |
|  | /ese/ | 108.08 | 125.18 | 114.33 | 347.59 | 36.01 |
|  |  | 113.23 | 111.83 | 109.62 | 334.68 | 33.41 |
|  |  | 105.94 | 109.98 | 119.22 | 335.14 | 32.82 |
|  | /esse/ | 109.68 | 266.12 | 123.65 | 499.44 | 53.28 |
|  |  | 120.14 | 236.54 | 120.58 | 477.26 | 49.56 |
|  |  | 120.02 | 237.45 | 113.13 | 470.60 | 50.46 |
|  | /iki/ | 105.96 | 84.37 | 123.38 | 365.10 | 23.11 |
|  |  | 82.02 | 81.80 | 74.29 | 277.67 | 29.46 |
|  |  | 84.08 | 105.93 | 123.77 | 355.03 | 29.84 |
|  | /ikki/ | 102.34 | 194.29 | 88.34 | 431.59 | 45.02 |
|  |  | 91.07 | 186.64 | 107.71 | 415.89 | 44.88 |
|  |  | 96.50 | 185.90 | 83.07 | 399.25 | 46.56 |
|  | /ipi/ | 97.94 | 115.03 | 118.80 | 346.93 | 33.16 |
|  |  | 96.78 | 117.53 | 121.61 | 347.81 | 33.79 |
|  |  | 105.34 | 96.53 | 99.26 | 314.27 | 30.72 |
|  | /ippi/ | 89.82 | 261.63 | 95.05 | 456.10 | 57.36 |
|  |  | 105.19 | 263.28 | 120.46 | 499.26 | 52.73 |
|  |  | 118.99 | 299.51 | 107.07 | 534.93 | 55.99 |
|  | /isi/ | 84.08 | 134.81 | 116.07 | 334.96 | 40.25 |
|  |  | 107.12 | 125.80 | 139.60 | 372.52 | 33.77 |
|  |  | 102.72 | 130.25 | 78.28 | 311.26 | 41.85 |
|  | /issi/ | 100.27 | 255.95 | 101.28 | 457.50 | 55.95 |
|  |  | 104.64 | 249.27 | 102.19 | 456.10 | 54.65 |
|  |  | 106.95 | 261.43 | 103.44 | 471.82 | 55.41 |

**Appendix Ⅲ.** The segment durations and CD/EWD values of each test word which used for sound synthesizing in experiment 2.

| Test word | Segment Durations (msec.) | | | EWD  (msec.) | CD/EWD  (%) |
| --- | --- | --- | --- | --- | --- |
|  | Preceding Vowel Duration | Constriction Duration | Following Vowel Duration |  |  |
| /akka/ | 111.87 | 263.58 | 128.83 | 520.23 | 50.67 |
| /appa/ | 103.91 | 270.01 | 142.53 | 522.40 | 51.69 |
| /assa/ | 145.57 | 298.02 | 134.21 | 577.80 | 51.58 |
| /ekke/ | 135.45 | 294.20 | 170.17 | 614.83 | 47.85 |
| /eppe/ | 109.87 | 304.58 | 133.29 | 554.49 | 54.93 |
| /esse/ | 120.17 | 275.06 | 141.04 | 536.27 | 51.29 |
| /ikki/ | 112.33 | 274.85 | 133.54 | 560.92 | 49.00 |
| /ippi/ | 107.08 | 284.01 | 139.97 | 540.92 | 52.50 |
| /issi/ | 111.14 | 334.59 | 132.99 | 578.72 | 57.82 |
